# Legal Status and Fertility Patterns: Regulation-Induced Disruption Among Previously Undocumented Immigrant Women in Italy

**DOI:** 10.1007/s10680-024-09707-5

**Published:** 2024-06-11

**Authors:** Rocco Molinari, Roberto Impicciatore, Livia Elisa Ortensi

**Affiliations:** Department of Statistical Sciences “Paolo Fortunati”, University of Bologna, via delle Belle Arti 41, 40126 Bologna, Italy

**Keywords:** Immigrant women, Fertility patterns, Irregular migration, Legal status, Italy

## Abstract

We explore, using a unique survey dataset containing retrospective information on immigrants’ legal status, the relationship between previous irregular experience—from arrival up to the first residence permit achievement—and fertility patterns among non-EU immigrant women in Italy. While competing hypotheses explaining migrants’ fertility behaviour have been recurrently offered, there is a substantial lack of knowledge on the role of undocumented experience as a contextual barrier in shaping international migrants’ family formation processes. We adopt a life-course approach, employing event history analysis and Poisson regression modelling, to investigate how irregularity among immigrant women intertwines with the timing of the first childbirth and the total number of births occurred in Italy. We find that irregular experience—as a time-dependent process—delays the transition to childbirth post-migration. Furthermore, having experienced irregular status reduces completed fertility, offering few possibilities to catch-up over the life-course with fertility levels of women continuously having the legal status. Findings suggest long-lasting effects of irregular status and the potential disruption of migrant’s fertility induced by migration policies, admission systems, and regulation factors. The reduced possibility of legal entry channels and lack of migration policies for planning and managing migration into Italy may thus have an impact on family formation trajectories among international immigrant women.

## Introduction

Migrants’ family dynamics are crucial for understanding the forms and trajectories of their integration. To what extent (and through which processes) immigrants change their demographic behaviour in destination contexts or maintain part of their traditions, ideologies, and practices are fundamental issues in studying geographical mobility patterns. Nonetheless, understanding the fertility behaviour of immigrant populations raises public and scholarly interest due to their potential contribution to (declining) fertility and ageing dynamics of many European receiving countries.

In the last decades, among scholars interested in the study of immigrants’ fertility, a shift has emerged from the investigation of aggregate-level indicators to the analysis of individual-level patterns in a life-course perspective (Kulu et al., 2019). The initial focus on the quantum of immigrants’ fertility and its contribution to the TFR has been matched with the interest in the timing of post-migration fertility trajectories (Andersson, 2004; Sobotka, 2008; Toulemon, 2004). Such an approach has allowed scholars to analytically consider several individual trajectories of immigrants (e.g. couple formation, marriage, living arrangements) and investigate how they intertwine with the timing of births (Clark et al., 2009; Cooke, 2008; Landale, 1997). However, most previous research, especially in Europe, has been carried out on immigrants currently having the right to reside in destination countries. Therefore, among factors shaping international migrants’ fertility trajectories, the role of undocumented experience as the result of a regulation-induced contextual barrier has remained in the background (Bean & Brown, 2014).

This lacuna appears particularly relevant for studying Southern European contexts of reception, which for the last forty years—also due to their well-developed informal economy (Reyneri, 2001, 2003; Triandafyllidou, 2013)—have become targets for strong migratory inflows. Irregular migration flows have been highly tolerated in Southern Europe, and recurrent ex-post regularisation programmes have stabilised the rapidly expanding migrant population (Colombo & Dalla Zuanna, 2019; King & Okólski, 2018; Colombo, 2012). However, while irregularity has been considered the first step in a career of settlement and ‘citizenization’ (Ambrosini, 2015; Glytsos, 2005), the medium and long-term consequences of experiencing irregularity on migrants’ life-course trajectories have been largely unexplored.

In this study, we analyse the relationship between individual legal status trajectories and fertility patterns among international immigrants in Italy adopting a life-course approach. Notably, we aim to investigate how previous irregular experience (and its duration) is associated with both tempo and quantum fertility among immigrant women in Italy. Our analysis contributes to current knowledge of the fertility behaviour of migrants in several ways. First, we provide fresh evidence on the role of legal status in shaping fertility patterns of migrant women. Previous studies (almost entirely conducted in the U.S.) have already shown that irregular status affects a variety of immigrant outcomes, from labour market to health conditions (Hall & Greenman, 2015; Kossoudji & Cobb-Clark, 2002; Zajdel, 2023). However, except for a few recent works on the fertility consequences of legalisation and enforcement policies (Amuedo-Dorantes & Arenas-Arroyo, 2021; Amuedo-Dorantes et al., 2023), limited evidence exists on the relationship between legal status and family dynamics both in the U.S. and Europe.

Second, in our study the timing of irregular experience is matched with the timing of fertility. Although a dynamic approach to legal status has received some attention in the U.S. context (Jasso et al., 2008; Kreisberg, 2019), the study of the consequences of previous undocumented experience on international immigrants’ life-course outcomes is almost non-existent in Europe, mainly due to substantive data limitations. The retrospective information collected in the *Social Condition and Integration of Foreign Citizens* (SCIF) survey represents an unprecedented opportunity to observe the previous undocumented histories of immigrants, from their arrival to Italy to the first residence permit attainment. This allows us to distinguish between migrant women continuously experiencing regular status and previously undocumented women and observe irregular status duration. Such a retrospective approach to legal status appears particularly suited for investigating the Italian case and, more generally, the ‘Southern European model’ of migration (King & DeBono, 2013), so much distinguished by the accumulation of irregular migration stocks and ex-post regularisations. Although our analysis does not explicitly address legislation changes, such an investigation also offers the opportunity to delve into the role of contextual factors shaping migrant fertility patterns by examining how legislation translates into legal status trajectories at the individual level.

Finally, thanks to the availability of representative survey data, we offer a comprehensive analysis of fertility patterns among non-EU immigrant women in Italy. Previous studies on the Italian case have already stressed the importance of the interrelation between migratory patterns in terms of channels of admittance (e.g. family, work, and others) and fertility patterns (Mussino & Strozza, 2012a, 2012b). However, facing constraints due to the use of registry data, these analyses have nearly always restricted their observation to the time spent as regular migrants. In our study, we extend the observation window, shedding light on irregular pathways that have previously remained in the shadow, contributing to current knowledge on the relationship between fertility and migratory patterns.

The paper is structured as follows: Sect. 2 reviews the theoretical and empirical backgrounds in the study of immigrant women’s fertility patterns; Sect. 3 describes the Italian context, with an emphasis on female immigration processes; Sect. 4 explores the existing evidence on the relationship between legal status and immigrants’ outcomes and rises some methodological issues; Sect. 5 outlines our research hypotheses; Sect. 6 presents data and methods; Sect. 7 shows our findings; and Sect. 8 concludes.

## Fertility Patterns Among Immigrant Groups: Theoretical Underpinnings and Literature Review

Many competing and partially non-exclusive hypotheses have been offered, primarily by demographers, for the comprehension of both the quantum and tempo of fertility patterns among immigrant groups to the present day (Kulu et al., 2019; Bohon & Conley, 2015; Adserà & Ferrer, 2015; Kulu & Gonzalez-Ferrer, 2014; Kulu & Milewski, 2007). A first group of hypotheses has been focusing in understanding how the experience of geographical mobility (usually intended as a unique life-course event) impacts demographic behaviour in terms of change or resistance to change. Fertility patterns of immigrants have been explained in the framework of *socialisation* (Coleman, 1994; Herviz, 1985), *adaptation* (Gordon, 1964; Singley & Landale, 1998), and *selection* (Hoem, 1975; Macisco et al., 1970).

Empirical evidence suggests that fertility patterns among natives and immigrants from high fertility settings—so far, the most researched (Mussino et al., 2021)—differ systematically, the latter generally showing higher birth rates. This pattern has been extensively observed, e.g. among Mexican migrants in the U.S. (Bean et al., 2000; Choi, 2014) and Turkish women in Germany (Milewski, 2010a, 2010b). Furthermore, in some cases, native-immigrant gaps persist across second generations (Milewski, 2007, 2010a; Scott & Stanfors, 2011; Wilson, 2019). Studies investigating the characteristics of receiving contexts usually refer to the role of residential segregation in shaping socialisation into a ‘minority subculture’ among immigrant descendants (Hill & Johnson, 2004; Wilson & Kuha, 2018). Nonetheless, fertility patterns of international immigrants and their descendants are also likely to be shaped by other contextual factors, e.g. employment, occupations, and welfare (Dupray & Pailhé, 2018; Lundström & Andersson, 2012). However, how the characteristics of the context of reception shape migrants’ fertility trajectories has received little attention (Milewski & Adserà, 2023). Recently, comparative studies on multiple groups in multiple destinations have also addressed the role of receiving contexts in hindering or favouring the adaptation of immigrants, throughout settlement, to the fertility levels of destination countries (Kulu et al., 2017; Milewski, 2011; Mussino & Cantalini, 2022).

Other hypotheses on immigrant fertility refer to the specific consequences of geographical mobility (intended in its relationship with other life-course events) on the timing of births. The *interrelation of events* hypothesis considers how migration is mainly intertwined with other important family patterns, like couple formation, marriage, and reunion, which imply a fertility peak soon after migration and a subsequent downturn (Andersson, 2004; Lindstrom & Giorguli-Saucedo, 2007). In this vein, a recurrent finding—also observed in the Italian context—suggests that fertility patterns are usually interrelated with migratory patterns, meant as a channel of admittance or reason for migration alike. Empirical evidence has shown that family migrant women (i.e. those accessing a residence permit for family reasons) are more likely to experience fast transitions to childbearing due to the interrelation of migration with other family formation processes. By contrast, women migrating for work do experience lower transition rates. The relevance of migratory patterns in shaping the fertility behaviour of women usually persists even when citizenship is taken into account in the analyses (Mussino & Strozza, 2012a, 2012b; Ortensi, 2015; Mussino et al., 2015; Castro-Martín & Rosero-Bixby, 2011; Nedoluzhko & Anderrson, 2007).

An explicit focus on the timing also pertains to the *disruption* hypothesis, which assumes that migrants, immediately following migration events, show particularly low fertility levels due to the disruptive factors associated with geographical mobility. Indeed, migration usually implies separation from close relationships (including partner), psychological stress, and economic uncertainty, which lead to temporary postponement of childbearing until the conditions of family formation are met (Hervitz, 1985; Kahn, 1994; Mayer & Riphahn, 2000). Among factors inducing disruption, especially in Southern European countries, the national legislation, setting the conditions under which migrants are authorised to access and reside in the country, along with rules allowing undocumented migrants to come out of irregularity, may play a fundamental role (see Fox-Ruhs et al., 2024 for an extensive discussion of an institutional approach to understanding the conditions of irregular migrants in Europe). Irregular status prevents migrants from enjoying a wide range of fundamental rights and exposes them to the risk of deportation, exploitation and vulnerability. Irregularity can, therefore, be seen as a relevant factor inducing disruption in the migrants’ life course, which is likely to have a significant impact on their family dynamics.

Despite the relevance of the issue, most studies on the relationship between fertility and migration have been exclusively drawn from the analysis of migrants with regular status. In the study of fertility and family formation processes, no research has extended the investigation of the role of women’s migratory trajectory to periods of irregularity so far. Nonetheless, even migratory channels reflect different exposure to the risk of experiencing irregularity in migrants’ initial settlement. Family migrants typically access destination countries using legal admission channels through family reunification with their partner or parents. Economic migrants, on the contrary, especially in Southern Europe, have been predominantly exposed to irregularity. What is the role of legal status trajectories, independently from other factors, in shaping immigrant women’s fertility patterns?

## The Italian Context of Female Migration

In the last decades, Italy has been crossed by multiple and diverse female migratory inflows. The presence of international immigrant women in Italy has achieved some importance between the 1950s and 1970s (Colucci, 2018). In that early phase, a limited number of women—mainly from Eritrea, the Philippines, Cape Verde Islands, El Salvador, Somalia, and Sri Lanka—came to Italy to work as live-in housekeepers in middle-class families, partly thanks to connections between Catholic missions in origin areas and parishes in the destination (Andall, 2020; Tognetti Bordogna, 2023). However, more intense female migratory inflows have been observed since the 1990s and, especially, in the first decade of the 2000s, when family reunification began to rise substantially. Moreover, throughout these decades, Italy has become the destination of different and far more sizable flows of first migrant women from Latin America, the Philippines, and predominantly Eastern Europe, as a response to the growing labour demand in the domestic and care work sector (Catanzaro & Colombo, 2009; Sciortino, 2004).

Due to the significant delay in defining systematic immigration policies, since the early 1980s, Italy has recurrently issued several ex-post amnesties to legalise current undocumented migrants.^1^ Regularisation programmes were developed in 1982, 1986, 1990, 1995, 1998, 2002, 2009, 2012, and 2020. Previous estimates have shown that almost 3 million immigrants (both males and females) currently living in Italy have experienced irregular status during their settlement (Buonomo & Paparusso, 2018). Among non-EU immigrant women in Italy, the share of migrants with previous irregular status has been estimated at more than 50 per cent (Molinari et al., 2023). Therefore, despite the relevance of family reunification as a legal channel of admission, a large size of immigrant women have been previously involved in irregular spells before accessing legal status.^2^

Labour market participation of first-generation migrant women in Italy has remained highly differentiated by national origin. Women from Latin America, Eastern Europe, China, and the Philippines have generally higher employment rates than Italian women, mainly working in sales activities, restaurants and hotels, and personal services. For example, among Ukrainian and Moldovan women, three out of four migrated for work reasons (Buonomo et al., 2020). Conversely, women from North Africa, the Middle East, and the Indian subcontinent generally participate less in the labour market, mainly being family dependents.

Due to the multiple composition of female migratory flows, different immigrant fertility models coexist in Italy (Gabrielli et al., 2019; Impicciatore et al., 2020; Mussino & Strozza, 2012a). Family migrants have a higher overall number of births and a higher intensity of transition to the first birth after migration than female pioneers who migrated for work. Consistently, high birth rates of Moroccan and Albanian women in Italy have been explained in terms of interrelation of events and socialisation. In contrast, lower fertility of Ukrainian and Romanian women has been mainly referred to as disruption and selection processes.^3^

Voluntary abortion among foreign women in Italy is higher compared to native women, but the incidence is decreasing over time. Evidence has shown that abortion rates among Romanian women dropped from 7.2 per cent in 2003 to 2.7 per cent in 2011, following the post-enlargement entering of Romania into the EU in 2007 and the de-facto regularisation of all irregular Romanian women in Italy (D’Errico et al., 2017). While a reduction in abortion rates was observed for many (but not all) foreign-born women between 2003 and 2011, no other groups had a similar drop, suggesting a role of irregular status in creating an incompatibility with childbearing.

## Legal Status and Immigrants’ Outcomes: Evidence and Methodological Issues

In recent years, some studies—mainly based in the U.S. —have investigated the relationship between legal status and a variety of immigrants’ social integration outcomes: from occupations and wages to employment, from health to crime, from naturalisation propensities to remittances. Researching irregular migration poses relevant methodological challenges, spanning from identification issues to a substantial lack of information (Strozza, 2004). On the one hand, large nationally representative surveys including immigrants in their sampling design, if drawn on the population registry, only include respondents having the right to reside in the destination country. On the other hand, large destination-centred surveys of international immigrants do not generally collect information on respondents’ visas and residence permits. In short, surveys including information on immigrant legal status are usually small and address specific migration sub-groups (Bachmeier et al., 2014; Serrano Sanguilinda et al., 2017).

Therefore, research studies on the consequences of irregular migration have usually followed two different empirical approaches. First, using existing available survey data with information on visas and residence permits, some scholars have adopted a cross-sectional approach comparing outcomes of undocumented immigrants with those having legal status at the interview date, i.e. without considering previous experiences. In some cases, imputation methods have been applied to extensive U.S. surveys to infer legal status through observable characteristics (Passel & Cohn, 2014; Warren, 2014). Findings reveal that irregular immigrants face substantive labour market disadvantages in terms of poor wage returns, occupational penalties, and risks of being trapped in hazardous jobs (Hall et al., 2019; Borjas & Cassidy, 2019; Hall & Greenman, 2015).

Second, exploiting legalisation programmes as exogenous policy changes, other studies have investigated the consequences of legal status attainment, i.e. the transition from undocumented to documented status.^4^ Empirical evidence, with some exceptions, has shown that legal status transition positively affects wages while reducing employment (Fasani, 2015; Kossoudji & Cobb-Clark, 2002; Monras et al., 2020), fosters migrants’ consumption behaviour (Dustman et al., 2017), and reduces risks to commit a crime (Pinotti, 2017). The legal-status-achievement effect has also been observed in relation to remittances (Amuedo-Dorantes & Mazzolari, 2010) and schooling (Amuedo-Dorantes & Antman, 2017; Felfe et al., 2020).

Recently, the policy change approach has been applied to the study of migrants’ fertility as well. Amuedo-Dorantes and colleagues (2023) have shown that the introduction of an immigration policy in Spain granting legal status to mothers of children with Spanish citizenship increased the childbearing of eligible mothers. Other immigration policy changes are likely to have specific effects on undocumented migrants’ fertility, as in the case of immigration enforcement initiatives in the U.S. lowering childbearing of undocumented immigrant women (Amuedo-Dorantes & Arenas-Arroyo, 2021).

Despite its relevance, the current-status and policy-change approaches mainly offer short-term assessments of lacking legal status, failing to comprehensively evaluate the long-term consequences of prolonged irregularity and legal status changes on immigrants’ trajectories. Nonetheless, previous undocumented experiences, through time, are likely to leave traces in the life course of immigrants even after legalisation, shaping subsequent patterns. Recently, some studies have applied a longitudinal approach to modelling the legal status as a dynamic category for the comparison of migrants who have experienced irregular spells before achieving legal status and those who have continuously experienced legal conditions (Shoumaker et al., 2022; Cheong, 2021; Jasso, 2011; Jasso et al., 2008). Previously undocumented immigrants have been observed to obtain less prestigious jobs in the long run (Kreisberg, 2019; Molinari et al., 2023) and to experience persistent disadvantages in health outcomes (Zajdel, 2023). Despite its relevance for investigating receiving contexts where undocumented experiences are widespread—e.g. Southern European countries—this approach has received limited attention.

More generally, the empirical investigation of legal status and immigrant outcomes is particularly scarce among European countries. First, the difficulty of reaching irregular migrants and the lack of information on the (current or previous) legal status in Europe have severely limited this research strand. Second, studies in European countries have almost entirely focused on immigrant economic outcomes in terms of labour market and consumption (Baldacci et al., 1999; Fasani, 2015; Dustman et al., 2017; Devillanova et al., 2018), overlooking the impact of irregular status on other non-strictly economic aspects of immigrant lives. Finally, they are overly unbalanced towards other forms of legal status acquisition—e.g. EU citizenship, refugee status, or other types of legal status based on motivation (Fellini & Guetto, 2022; Ruhs, 2017; Zwysen, 2019)—largely ignoring the problem of the absence of the legal right to reside in the destination country. Due to this complex set of factors, the investigation of the relationship between irregularity and family dynamics has remained in the background in Europe. However, irregularity involves a considerable size of migrants among European countries and many undocumented migrants living in the EU are women of reproductive age (PICUM, 2016). Previous figures estimated that in 2008 between 1.9 and 3.8 million undocumented migrants were living in the EU28 (Vogel et al., 2011). More recent estimates report that in 2017 undocumented migrants might vary between a maximum of 4.8 and a minimum of 3.9 million in the former EU28 plus four EFTA countries (Connor & Passel, 2019). In Italy, 519 thousand irregular immigrants (9 per cent of the entire foreign population) were estimated to reside in 2019 (ISMU, 2022).

## Research Hypotheses

As already noticed in a previous study by Falasco and Heer (1984), legal status is likely to affect fertility patterns of migrants both directly and indirectly. Firstly, irregular migrants are usually constrained to low-wage and underqualified jobs, facing higher risks of downward mobility patterns over time. Lacking legal status is, therefore, largely associated with economic uncertainty, which in turn may hinder migrants’ fertility. Furthermore, irregular migrants have limited or no access to essential services for their settlement (e.g. credit, housing, and welfare), they are not allowed to travel back and forth to their origin country and run high risks of incarceration and deportation. In short, irregular immigrant women face high psychological stress due to their condition, which is likely to have disruptive implications on fertility patterns (Paparusso et al., 2017). Secondly and more directly, legal status may influence migrants’ fertility by providing irregular immigrant women with limited access to maternal public healthcare during pregnancy and no needed support for the child (and the mother herself) after birth (e.g. medical assistance and childcare services). Therefore, our first hypothesis is the following:

### Hp1

(Tempo Hypothesis) Among immigrant women in Italy, irregular status is associated with a delayed transition to the first post-migration birth, accounting for other socio-demographic characteristics and migratory factors.

Furthermore, previous irregular histories, due to disruption associated with their duration, may maintain long-lasting effects, which are likely to influence not only the timing but also the overall fertility of immigrant women in Italy, hindering the possibility of a fertility catch-up even after the legal status achievement:

### Hp2

(Quantum Hypothesis) Previously undocumented immigrant women in Italy, compared to continuously legal ones, experience lower fertility post-migration, accounting for other socio-demographic characteristics and migratory factors.

## Data and Methods

To investigate these aspects, we use data provided by the *Social Condition and Integration of Foreign Citizens* (SCIF) survey, collected by the Italian National Institute of Statistics (ISTAT) in 2011–12 on a representative sample of the foreign population in Italy.^5^ The sampling design is based on households identified through the civil registry (thus having the legal right to stay in Italy at the interview date) in which one member at least is a foreign national. Within each household, all members have been separately interviewed (*N* = 25,326).

The sub-sample used in our analysis includes only non-EU immigrant women who first arrived in Italy in their reproductive age (18–36 years), childless or with one child born before migration at most, between 1989 and 2012. It thus consists of first-generation immigrant women who were required to have a visa or a resident permit when they first accessed Italy (*N* = 2,430). EU national respondents at the survey date (2012) are excluded. Indeed, the SCIF survey collected retrospective information on the first residence permit (which are used to construct our measures of irregularity) only for currently non-EU nationals.

Although based on currently regular immigrant respondents, the SCIF survey retrospectively collected extensive information on the first residence permit, including the type of permit and the date of achievement. By combining this information with the timing of the migration trajectory, we were able to investigate previous time spent as undocumented between the access into Italy and the first permit. Unfortunately, the SCIF data do not include information on immigrants’ legal trajectory following the first permit. Therefore, our analysis focuses exclusively on the initial irregular experiences without considering subsequent relapses into undocumented status. Furthermore, the survey retrospectively collected all women's birth events.

As the primary independent variable, we consider two measures of previous irregular experience. First, we define *time spent in irregular status* as a time-constant variable recoded into three categories. The first one is labelled *continuously legal* and refers to female respondents for whom the three following conditions are met simultaneously: (1) she has never held irregular status (determined by answering a direct question); (2) she had not obtained the first residence permit through a regularisation programme (which by definition implies irregular status); and (3) the year of obtaining the first permit is the same or, at most, one year later than arrival. The other categories—*0–1 years*; *2* + *years*—identify the previous irregular experience of immigrant women based on the length of time from entry to the first residence permit.

As a second measure of previous irregular experience, we compute the time-varying dummy variable *currently irregular,* labelled as 0 while the immigrant woman is experiencing regular status and as 1 while she is experiencing irregular status. Thus, for migrants continuously having the legal status (i.e. the first category of time spent in irregular status), currently irregular is always set to 0. This variable, used for hazard models of fertility patterns (see below), is based on the dates of access into Italy and first permit attainment.

It should be noted that we identify irregular status irrespective of the type of access into Italy, which can be authorised or unauthorised. It is well known that many immigrants who accessed Italy legally (typically holding a tourist visa) have become overstayers after a few months. However, due to the lack of available information on visas in the SCIF data, we do not consider visa overstayers separately.

In our analysis, we also include several socio-demographic variables that are likely to affect fertility patterns of immigrant women: *place of birth* (as a single country or aggregation of countries of the same geographic area), which reflects the heterogeneity of female immigrant origins in Italy (Ukraine, Other Eastern Europe, China, Other Asia, Morocco, Other MENA, Sub-Saharan Africa, Latin America); *age on arrival* in Italy, in five-years categories (18–21, 22–26, 27–31, 32–36); *education* (No school and lower secondary, Upper secondary; Tertiary); *area of residence* in Italy (North-west, North-East, Centre, South and Islands) and, to account for different phases of Italian immigration history, *cohort of arrival* (1989–1998, 1999–2008, 2009–2012). Moreover, we include a dummy variable that accounts for having a *child pre-migration*. Finally, since previous studies have already observed the relationship between migratory channels and immigrant women’s fertility, we also consider the *type of first residence permit* (Work, Family, Other) as a proxy of the migratory channel, even though permit attainment may occur some years after migration. The univariate distribution of these variables is shown in the Appendix, Table 4.

**Fig. 1:**
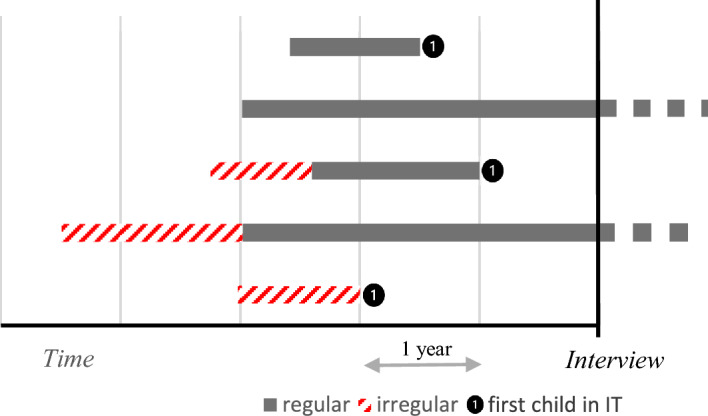
Representation of immigrant women’s fertility trajectories in Italy, based on observed mean durations of regular and irregular sub-episodes in different cases

We explore the relationship between legal status and fertility patterns in two ways. In the first step, we test, through an event history analysis (Blossfeld et al., 1989), whether the timing and risk of transition to the first child in Italy (post-migration) changes among immigrant women experiencing irregularity—e.g. while lacking legal status—compared to those experiencing regularity—e.g. while having the legal status. In other words, we estimate the impact of being currently undocumented on the propensity to have the first child in Italy. To address this issue, we develop a continuous-time Piecewise Constant Exponential hazard model considering the duration between the woman’s arrival in Italy and the first post-migration birth (if any) or the interview (if the woman has not given birth to any child in Italy before the survey). In the specification of the hazard model, we assume that the baseline function is constant over the following intervals: 0–3 years, 3–5 years, 5–10 years, 10 years or more. By including the time-varying variable *currently irregular*, we identify regular and irregular periods (or sub-episodes), and thus, we investigate the influence of patterns of legal status on the timing of birth. Figure 1 represents individual trajectories that may emerge in our data (based on observed mean durations of sub-episodes). Specifically, there are five possible combinations. Except for censored cases (women without children born in Italy), some women experienced the transition to first childbirth in Italy while continuously having legal status, other women while having legal status with a previous irregular episode, and other women as undocumented migrants.

In the second step, we investigate the relationship between previous irregular experience and post-migration overall fertility in Italy among immigrant women, to assess whether obtaining legal status is associated with a fertility catch-up irrespectively from the possible delay of lacking legal status. In this case, our dependent variable is the *total number of births* that occurred in Italy. Since our response variable involves count data, we apply a Poisson regression model to study the relationship between *time spent as an irregular migrant* and the *total number of births* using the number of fertile years spent in Italy (from the access up to 49 years of age) as an offset variable. This regression model assumes the response variable to have a Poisson distribution and the logarithm of its expected value can be expressed as a linear combination of covariates (see, e.g., Gabrielli & Giannantoni, 2015). By including the woman’s age of arrival among covariates and considering the exposure time up to 49 years, we control for the different age composition of respondents. As other independent variables, we include the same set of covariates used for hazard models.

## Results

Table 1 shows that, in our subsample, previously irregular women represent the majority (57 per cent) compared to women who have continuously experienced legal status (43 per cent). Even though the estimated percentage of immigrants with irregular status over total immigrants in Italy was around 10 per cent in 2011–2012 (ISMU, 2022), a significantly larger proportion of regularly residing immigrant women had gone through the undocumented experience. This result is in line with the Italian model of incorporation, largely based on the admission of unauthorised migrants (or visa overstayers) and the implementation of ex-post regularisations. Long-lasting irregular periods (more than 2 years) are the most widespread (38 per cent). Furthermore, previous irregular experience is associated with the type of first residence permit, thus indirectly with the migratory channel. Among women who accessed the first permit for employment reasons, those with previous undocumented experience count for almost 75 per cent. In contrast, previously irregular women are less than 50 per cent among family dependents. However, in both the types of first permits long-lasting irregular experiences (2 years or more) are the most widespread (46 per cent vs. 35 per cent). Therefore, besides the strict connection between employment permit attainment and irregular experience that characterises the Italian context, cases of lacking legal status among family migrant women are not uncommon, in some instances lasting several years.

**Table 1:** Time spent as irregular migrant by type of first residence permit for immigrant women

	Work	Family	Other	Total
Continuously legal	26.8	52.3	40.3	43.0
Irregular 0–1	26.7	12.9	26.8	18.5
Irregular 2 +	46.5	34.8	32.9	38.5
Tot	100	100	100	100

Weighted cases

**Fig. 2:**
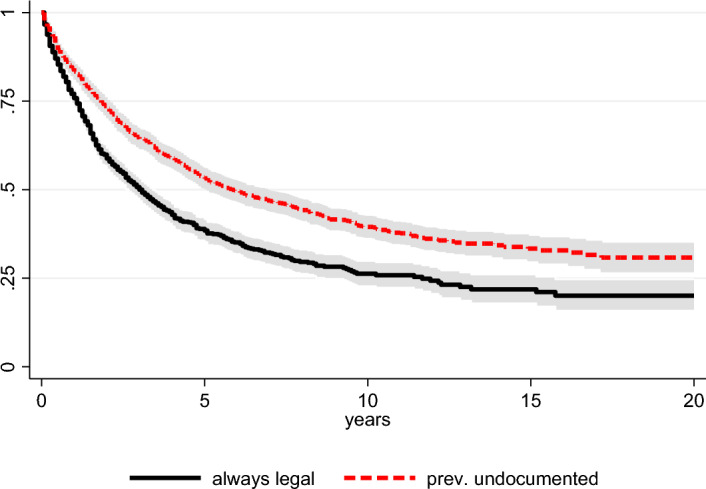
Kaplan–Meier survival estimates and 0.95 confidence intervals on the transition to the first child in Italy for immigrant women, by previous undocumented experience

Among non-EU immigrant women in our subsample, about 60 per cent (*N* = 1,435) experience a transition to the first child post-migration (failures). The pace of transition to first child in Italy (or first child ever for some cases) accelerates in the first four years post-migration, then starts declining constantly, showing an overall median duration of 4 years and 6 months. However, relevant differences in the timing of transition exist by previous irregular experience. Figure 2 shows Kaplan–Meier survival estimates (and 0.95 confidence intervals) of time to first child in Italy among immigrant women who have continuously held legal status since the access and those who have experienced a previous irregular spell (irrespectively from its duration), for the first 20 years since migration. Survival curves show that women continuously having the legal status have a faster transition to the first child than those with a previous irregular status, having a median duration to the event of 3.1 years versus 5.8 years.

However, other factors, which are supposed to be correlated with being an irregular migrant, are also likely to affect fertility patterns. Furthermore, legal status is likely to change over time defining episodes of irregularity in the individual life-course as a time-dependent process. Hence, we applied a multivariate Hazard model on the transition to the first child in Italy encompassing the time-varying variable denoting currently irregular status. Table 2 presents results for Model 1, without the type of first permit among covariates, and Model 2, including the type of first permit, to show whether differences exist between the two model specifications in the association between currently irregular status and transition to the first child. Estimates reveal that the time-dependent legal status trajectory is associated with the timing of transition to the first birth in Italy, in both models. *Ceteris paribus*, while immigrant women are in irregular status, between the access into Italy and the first permit achievement, the conditioned hazard of experiencing the event is about 20 per cent lower as compared to periods in regular status (hazard ratio: 0.82). The negative association between irregular status and fertility propensity holds even accounting for women’s migratory channel. As expected, female family migrants do experience a much faster transition to the first child in Italy as compared to women accessing employment permits, who show lower risks and delayed timing.

**Table 2:** Piecewise constant exponential model on the transition to the first child in Italy

	Model (1)		Model (2)	
	Hazard ratios	Std. err	Hazard ratios	Std. err
*t*_1_	0.35***	(0.039)	0.18***	(0.023)
*t*_2_	0.21***	(0.027)	0.12***	(0.016)
*t*_3_	0.15***	(0.019)	0.09***	(0.012)
*t*_4_	0.08***	(0.014)	0.05***	(0.008)
Current irregular status—TV (ref. Regular)				
Irregular	0.83***	(0.050)	0.82***	(0.050)
Place of birth (ref. Albania)				
Ukraine	0.27***	(0.040)	0.36***	(0.055)
Other Eastern Europe	0.60***	(0.061)	0.69***	(0.070)
China	0.69***	(0.093)	0.80*	(0.108)
Other Asia	0.71***	(0.066)	0.79***	(0.073)
Morocco	1.06	(0.096)	1.07	(0.097)
Other MENA	1.35***	(0.143)	1.19	(0.127)
Sub-Saharan Africa	0.68***	(0.073)	0.78**	(0.085)
Latin America	0.48***	(0.047)	0.53***	(0.052)
Education (ref. No school and lower sec.)				
Upper secondary	0.84***	(0.049)	0.85***	(0.050)
Tertiary	0.83**	(0.076)	0.90	(0.083)
Age on arrival (ref. 22–26)				
18–21	0.98	(0.064)	0.94	(0.061)
27–31	0.72***	(0.052)	0.73***	(0.053)
32–36	0.39***	(0.043)	0.44***	(0.048)
Area of residence (ref. North-West)				
North-East	1.17**	(0.092)	1.12	(0.088)
Centre	0.91	(0.075)	0.97	(0.079)
South and Islands	0.82***	(0.060)	0.82***	(0.060)
Cohort of arrival (ref. 1989–1998)				
1999–2008	1.12*	(0.072)	1.07	(0.069)
2009–2012	1.13	(0.147)	0.98	(0.128)
Child pre-migration (ref. Childless)				
1 Child	0.92	(0.060)	0.83***	(0.054)
First permit (ref. Work)				
Family			2.62***	(0.172)
Other/don't know			1.32**	(0.160)
N	2,430		2,430	
Failures	1,435		1,435	

^*^p < 0.10, **p < 0.05, ***p < 0.01

Among the other estimates included in Table 2, we notice that the timing and risk of transition to the first childbirth in Italy broadly change by immigrants’ place of birth, with Albanian, Moroccan, and Middle Eastern and Northern African women showing the fastest transition as compared to Latin Americans and, especially, Ukrainians confirming findings of previous studies. Age on arrival is also associated with fertility patterns post-migration; the lower the age, the higher the risk of transition to first child.

In the second stage of our multivariate analysis, we applied Poisson regression on the total number of births to investigate whether this negative association also persists in terms of a reduced number of births. As for transition models, Table 3 shows the resulting estimates by applying two model configurations: without and with migratory channel among covariates (respectively, in Model 3 and 4).

**Table 3:** Poisson regression models on the total number of births that occurred in Italy

	Model (3)		Model (4)	
	Incidence rate ratios	Std. err	Incidence rate ratios	Std. err
Time spent in irregular status (ref. Always legal)				
Irregular 0–1 year	0.86***	(0.051)	0.97	(0.059)
Irregular 2 + years	0.85***	(0.041)	0.90**	(0.044)
Place of birth (ref. Albania)				
Ukraine	0.36***	(0.048)	0.44***	(0.060)
Other Eastern Europe	0.74***	(0.063)	0.82**	(0.070)
China	1.04	(0.106)	1.16	(0.118)
Other Asia	0.86*	(0.066)	0.93	(0.072)
Morocco	1.28***	(0.092)	1.27***	(0.091)
Other MENA	1.51***	(0.120)	1.43***	(0.114)
Sub-Saharan Africa	1.00	(0.084)	1.09	(0.092)
Latin America	0.61***	(0.052)	0.67***	(0.057)
Education (ref. No school and lower sec.)				
Upper secondary	0.93	(0.044)	0.93	(0.044)
Tertiary	0.90	(0.069)	0.95	(0.074)
Age on arrival (ref. 22–26)				
18–21	1.01	(0.052)	1.00	(0.051)
27–31	0.77***	(0.046)	0.78***	(0.046)
32–36	0.43***	(0.043)	0.47***	(0.047)
Area of residence (ref. North-West)				
North-East	1.10	(0.067)	1.08	(0.066)
Centre	0.88*	(0.059)	0.92	(0.062)
South and Islands	0.85***	(0.049)	0.87**	(0.050)
Cohort of arrival (ref. 1989–1998)				
1999–2008	1.53***	(0.074)	1.46***	(0.071)
2009–2012	1.59***	(0.185)	1.46***	(0.171)
Child pre-migration (ref. Childless)				
1 Child	0.91*	(0.048)	0.87***	(0.046)
First permit (ref. Work)				
Family			1.81***	(0.100)
Other/don't know			1.22**	(0.119)
Constant	0.11***	(0.010)	0.07***	(0.007)
Pseudo-R2	0.087		0.113	
*N*	2,430		2,430	

^*^p < 0.10, **p < 0.05, ***p < 0.01

Results of Model 3 show that having a previous irregular experience for immigrant women is generally associated with lower total fertility during settlement in Italy. Both short and long durations in irregular status are characterised by decreased total fertility, accounting for other factors.

However, once the type of first permit is included (Model 4), we notice that only women with long-lasting irregular spells (2 years or more) have lower fertility, experiencing a rate of children born in Italy that is 10 per cent lower than observed among women with continuously legal status. In short, our data suggest that when the length of irregular spells extends over 2 years, the ability of women to catch up with the number of births observed among women continuously having legal status is eroded, leaving permanent traces in migrant women’s biographies. However, women experiencing short irregular spells have higher chances of catching up with the initial delay.

We adopted some robustness checks in both the steps of our analysis. First, we developed Cox hazard models on the transition to childbirth, which do not require the definition of time intervals by leaving the baseline hazard unspecified. Coefficient estimates of these models—that are not shown but available upon request—confirm the results of Piecewise Constant Exponential models. Furthermore, to see whether estimates hold even considering only childless women at the entrance in Italy (thus excluding women with the first birth event before migration from our sub-sample), we run a duration model only for this subgroup. Estimates (shown in the Appendix, Table 5) largely confirm our findings. Second, since total fertility is better evaluated among older women and the age-composition at the interview may be affected by other covariates (including the time spent in irregular status), we run the Poisson regression model only among women with more than 36 years at the interview, also including age at the interview among covariates. Although relying on almost half of the sample size, estimates confirm that longer duration in irregular status is associated with lower completed fertility (results shown in the Appendix, Table 6). Finally, to ensure the robustness of our findings, we also fit the Conway-Maxwell-Poisson model for low fertility underdispersed count data (Barakat, 2017; Harris et al., 2012). Estimates for this model, which are not shown but are available upon request, confirm our results.

## Conclusions

This study focuses on the role of the time spent as an undocumented migrant as a potential barrier to childbearing among migrant women in Italy. Our results clearly highlight that an irregular status is associated with a delayed transition to the first child and lower post-migration fertility, confirming both Hypotheses 1 and 2 (*Hp1* and *Hp2*).

Considering the conventional hypotheses in the literature explaining migrant fertility, the disparity between documented and undocumented women revealed in our analysis suggests that legal status extends beyond the potential effects of adaptation, socialisation, selection, and event interrelation. These approaches do not consider the entry conditions and institutional and regulatory contexts, which, as our research emphasises, are significantly associated with the life patterns of immigrants in the destination context. Conversely, the negative impact of the irregular status may be viewed as an amplification of a disruptive effect, likely linked to psychological stress, economic uncertainty and vulnerability, disadvantages and segregation in the labour market.

In recent years, numerous contributions have reevaluated the significance of regulating migration (see, for instance, Massey, 1999). Particularly after September 11 attacks, there has been a marked acceleration in political restrictions on mobility in the U.S. and subsequently in various European countries (de Haas et al., 2018).

The normative regulation of migration encompasses legislative production, governmental actions, law enforcement, territorial control capabilities, and the role of judicial systems. These factors, steered by political considerations, profoundly impact migrants’ lives, with consequences that endure over time. In the Italian case, imposed restrictions have significantly altered migration flows (Bonifazi, 2013), impeding movements explicitly tied to labour market demands and favouring alternative streams like family reunification, asylum, and humanitarian requests. The substantial reduction, or even de facto abolition, of work entry opportunities has favoured irregular migration. This outcome, to some extent, stems from regulatory control that classifies and treats migrant individuals differently: some as regular workers, others as regular but unauthorised for work, and yet others as irregular despite their integration into receiving countries’ economies in various ways (Ambrosini, 2018).

These considerations prompt us to acknowledge the disruption induced by regulatory factors, migration policies, and admission systems that can leave lasting imprints on individuals’ life courses. The absence of legal entry channels and effective migration policies for planning and managing migration into Italy may thus have an impact on family formation among migrants. In a country strongly characterised by very low fertility and a rapid ageing process, hampering the fertility of migrants does not seem to be a wise measure.

Nonetheless, our analysis presents some limitations worth reviewing. First, the validity of our results is exclusively referred to immigrant women who have achieved legal status at the interview date, seeing as currently irregular ones are excluded by the survey’s sampling design. Unfortunately, to our knowledge, no survey data exist at the national level in European countries that allow an investigation of previous irregular histories, including both currently undocumented and documented immigrants. Furthermore, in our study, we can only investigate irregular episodes from migration to the first permit achievement. Therefore, we do not consider relapses into irregularity because data to conduct such an exploration are currently unavailable for the Italian case. However, it can be argued that disruption in fertility behaviour may be even enhanced among women who have never acquired a document, staying in irregular status for many years. Further research is needed to shed light on the complexity of international immigrants’ legal status trajectories and investigate its relationship with family formation processes.

A second point relates to a potential reverse causation issue. Disentangling the causal mechanism in the association between irregular experience and childbearing is difficult. In our analysis, such a relationship can be interpreted as the (direct or indirect) effect of the process of irregularity in hindering women’s fertility. However, we cannot exclude that the opposite can also be argued: childbearing is also likely to be pursued as a means to achieve legal status. Migrant women may consider children as an attempt to ground their legal status to that of their children, thus legitimating their right to stay in destination contexts highly characterised by exclusionary barriers (Bledsoe et al., 2007; Decimo, 2021). As a matter of fact, in Italy, for the period considered in our analysis, irregular immigrant women, from the sixth month of pregnancy, could apply for a short-term residence permit that lasts up to the sixth month of the child. Therefore, a lower risk of birth events for irregular migrants in Italy could be interpreted as the consequence of legal status achievement for undocumented pregnant women who experience childbirth while having legal status. To account for this aspect, we run a duration model on the transition to pregnancy—nine months before the first child transition. Results confirm the delayed effect of irregular experience (see Table 7 in the Appendix). This suggests that periods of irregular status not only hinder birth events, but also affect the transition to pregnancy, when no legal status for undocumented migrant women could be granted. However, another problem in identifying causal mechanisms pertains to the possible role of selectivity in affecting previous irregular experiences. In our analytical framework, we can only identify women currently having legal status. As already stated, those women who have stayed in irregular status up to the interview are excluded from the sampling design. This aspect has implications in terms of return migration selectivity. It must be considered that irregular immigrants, failing to achieve legal status in the long run, could be more likely to re-migrate than migrants continuously having legal status. This could be the case for immigrant women having a child in Italy while being in irregular status. Therefore, excluding return migrant women in our analysis would imply an underestimation of the negative effect of irregular experience on fertility patterns.

Despite these limitations, we think that a reflection on the long-term consequences of regulation-induced disruption in migrants’ biographies (also once the legal status is achieved) is greatly needed in fertility and, in general, in migration studies.

## Data Availability

The micro-data analysed during the current study are available in the ISTAT repository as Scientific-use Files, that may be requested exclusively for carrying out specific research projects by researchers belonging to Entities recognised as research institutions by Comstat or included in the list of research Institutions recognised by Eurostat. https://www.istat.it/en/analysis-and-products/microdata-files. The ideas and the views expressed in this manuscript should not under any circumstances be regarded as stating an official position of ISTAT. The results and any errors are entirely the responsibility of the authors alone.

## Appendix

See Tables

**Table 4:** Sample description

	*N*	%
*Place of birth*		
Albania	334	13.75
Ukraine	195	8.01
Other Eastern Europe	328	13.49
China	135	5.56
Other Asia	379	15.61
Morocco	306	12.59
Other MENA	157	6.47
Sub-Saharan Africa	223	9.17
Latin America	373	15.35
*Education*		
No school and lower sec	934	38.43
Upper secondary	1160	47.73
Tertiary	336	13.84
*Age on arrival*		
18–21	708	29.12
22–26	933	38.4
27–31	523	21.51
32–36	267	10.97
*Area of residence*		
North-West	901	37.09
North-East	678	27.91
Centre	531	21.86
South and Islands	319	13.14
*Cohort of arrival*		
1989–1998	499	20.53
1999–2008	1697	69.82
2009–2012	234	9.65
*First residence permit*		
Work	772	31.76
Family	1462	60.17
Other/don't know	196	8.07
*Child pre-migration*		
Childless	1810	74.49
1 child	620	25.51

Weighted cases

4,

**Table 5:** Piecewise constant exponential model on the transition to the first child in Italy, only childless women on arrival

	Hazard ratios	Std. err
*t*_1_	0.19***	(0.026)
*t*_2_	0.12***	(0.019)
*t*_3_	0.09***	(0.014)
*t*_4_	0.06***	(0.012)
Current irregular status—TV (ref. Regular)		
Irregular	0.80***	(0.056)
Place of birth (ref. Albania)		
Ukraine	0.37***	(0.064)
Other Eastern Europe	0.62***	(0.073)
China	0.77	(0.123)
Other Asia	0.70***	(0.073)
Morocco	0.99	(0.102)
Other MENA	1.05	(0.131)
Sub-Saharan Africa	0.70***	(0.089)
Latin America	0.48***	(0.053)
Education (ref. No school and lower sec.)		
Upper secondary	0.89*	(0.060)
Tertiary	0.91	(0.096)
Age on arrival (ref. 22–26)		
18–21	0.94	(0.067)
27–31	0.77***	(0.068)
32–36	0.46***	(0.065)
Area of residence (ref. North-West)		
North-East	1.19*	(0.106)
Centre	0.95	(0.091)
South and Islands	0.89	(0.074)
Cohort of arrival (ref. 1989–1998)		
1999–2008	1.00	(0.074)
2009–2012	0.87	(0.133)
First permit (ref. Work)		
Family	2.78***	(0.209)
Other/don't know	1.31**	(0.179)
*N*	1,785	
Failures	1,089	

^*^*p* < 0.10, ***p* < 0.05, ****p* < 0.01

^5^,

**Table 6:** Poisson regression models on the total number of births that occurred in Italy, only for women aged 36 or more at the interview

	Incidence rate ratios	Std. err
Time spent in irregular status (ref. Always legal)		
Irregular 0–1 year	0.88	(0.079)
Irregular 2 + years	0.87**	(0.061)
Place of birth (ref. Albania)		
Ukraine	0.50***	(0.090)
Other Eastern Europe	0.92	(0.123)
China	1.32*	(0.189)
Other Asia	1.02	(0.123)
Morocco	1.42***	(0.163)
Other MENA	1.54***	(0.198)
Sub-Saharan Africa	1.18	(0.147)
Latin America	0.80*	(0.100)
Education (ref. No school and lower sec.)		
Upper secondary	1.01	(0.069)
Tertiary	0.98	(0.105)
Age (ref. 36–39)		
40–44	0.73***	(0.052)
45 +	0.47***	(0.048)
Area of residence (ref. North-West)		
North-East	1.08	(0.096)
Centre	0.92	(0.086)
South and Islands	0.85**	(0.070)
Cohort of arrival (ref. 1989–1998)		
1999–2008	1.07	(0.072)
2009–2012	0.89	(0.367)
Child pre-migration (ref. Childless)		
1 Child	0.79***	(0.056)
First permit (ref. Work)		
Family	1.74***	(0.127)
Other/don't know	1.27*	(0.181)
Constant	0.08***	(0.011)
Pseudo-R2	0.113	
*N*	1,127	

^*^*p* < 0.10, ***p* < 0.05, ****p* < 0.01

^6^ and

**Table 7:** Piecewise constant exponential model on the transition to pregnancy in Italy

	Hazard ratios	Std. err
*t*_1_	0.19***	(0.024)
*t*_2_	0.12***	(0.017)
*t*_3_	0.08***	(0.012)
*t*_4_	0.04***	(0.007)
Current irregular status—TV (ref. Regular)		
Irregular	0.85***	(0.053)
Place of birth (ref. Albania)		
Ukraine	0.39***	(0.060)
Other Eastern Europe	0.62***	(0.068)
China	0.81	(0.114)
Other Asia	0.79**	(0.078)
Morocco	1.07	(0.104)
Other MENA	1.07	(0.122)
Sub-Saharan Africa	0.82*	(0.094)
Latin America	0.58***	(0.059)
Education (ref. No school and lower sec.)		
Upper secondary	0.90*	(0.056)
Tertiary	0.95	(0.093)
Age on arrival (ref. 22–26)		
18–21	0.99	(0.068)
27–31	0.72***	(0.056)
32–36	0.44***	(0.051)
Area of residence (ref. North-West)		
North-East	1.17*	(0.097)
Centre	0.98	(0.086)
South and Islands	0.85**	(0.065)
Cohort of arrival (ref. 1989–1998)		
1999–2008	0.92	(0.061)
2009–2012	0.51***	(0.084)
Child pre-migration (ref. Childless)		
1 Child	0.89*	(0.063)
First permit (ref. Work)		
Family	2.35***	(0.160)
Other/don't know	1.16	(0.149)
*N*	2,346	
Failures	1,227	

^*^*p* < 0.10, ***p* < 0.05, ****p* < 0.01

^7^

## Footnotes

1 In some cases, amnesties were targeted to immigrant women. In 1995 the Dini Decree issued the regularisation to spouses for family reunification, whereas in 2009 the Law on disposal on public security was directed to immigrants working without permission in the care and domestic sector (see Paparusso et al., 2017).

2 Authors’ elaborations on ORIM survey data, exclusively referred to the Lombardy region, show that in the period 2001–2014 irregular immigrant women currently living in Italy represented, on average, 13.4 per cent of the total female immigrant population (with recurrent yearly fluctuations), whereas men were 19 per cent. Data are not shown, but are available upon request.

3 Estimates of the adjusted TFR following the method proposed by Toulemon and Mazuy (2004) with the SCIF data have been provided by Giannantoni et al. (2018), showing that Moroccan, Albanian, Ukranian, and Romanian immigrant women have a TFR of 2.28, 1.66, 1.22, and 1.28, respectively.

4 Generally, these studies are based on ad hoc surveys of applicants to regularisation programmes introduced in the U.S., most notably, the 1986 Immigration Reform and Control Act (IRCA) and, to a lesser extent, the 1997 Nicaragua Adjustment and Central American Relief Act (NACARA). In Europe, studies exist on the amnesties introduced in Italy by the 2002 ‘Bossi-Fini’ law and in Spain by the 2005 Normalisation Programme.

5 Further details available at https://www.istat.it/en/archivio/191097.
